# Fibroblasts Accelerate Formation and Improve Reproducibility of 3D Cellular Structures Printed with Magnetic Assistance

**DOI:** 10.34133/2020/3970530

**Published:** 2020-07-23

**Authors:** Sarah Mishriki, Srivatsa Aithal, Tamaghna Gupta, Rakesh P. Sahu, Fei Geng, Ishwar K. Puri

**Affiliations:** ^1^School of Biomedical Engineering, McMaster University, Hamilton, Ontario, Canada; ^2^Department of Mechanical Engineering, McMaster University, Hamilton, Ontario, Canada; ^3^Department of Materials Science and Engineering, McMaster University, Hamilton, Ontario, Canada; ^4^Walter Booth School of Engineering Practice and Technology, Hamilton, Ontario, Canada

## Abstract

Fibroblasts (mouse, NIH/3T3) are combined with MDA-MB-231 cells to accelerate the formation and improve the reproducibility of 3D cellular structures printed with magnetic assistance. Fibroblasts and MDA-MB-231 cells are cocultured to produce 12.5 : 87.5, 25 : 75, and 50 : 50 total population mixtures. These mixtures are suspended in a cell medium containing a paramagnetic salt, Gd-DTPA, which increases the magnetic susceptibility of the medium with respect to the cells. A 3D monotypic MDA-MB-231 cellular structure is printed within 24 hours with magnetic assistance, whereas it takes 48 hours to form a similar structure through gravitational settling alone. The maximum projected areas and circularities, and cellular ATP levels of the printed structures are measured for 336 hours. Increasing the relative amounts of the fibroblasts mixed with the MDA-MB-231 cells decreases the time taken to form the structures and improves their reproducibility. Structures produced through gravitational settling have larger maximum projected areas and cellular ATP, but are deemed less reproducible. The distribution of individual cell lines in the cocultured 3D cellular structures shows that printing with magnetic assistance yields 3D cellular structures that resemble *in vivo* tumors more closely than those formed through gravitational settling. The results validate our hypothesis that (1) fibroblasts act as a “glue” that supports the formation of 3D cellular structures, and (2) the structures are produced more rapidly and with greater reproducibility with magnetically assisted printing than through gravitational settling alone. Printing of 3D cellular structures with magnetic assistance has applications relevant to drug discovery, lab-on-chip devices, and tissue engineering.

## 1. Introduction

Three-dimensional (3D) cellular structures representing tumor models provide more physiologically relevant research data than from two-dimensional (2D) cell cultures. These 3D models exploit *in vivo* cellular phenomena such as cell-cell interactions, cell polarization, increased drug resistance, and diffusion gradients of O_2_, CO_2_, nutrients, and metabolites that lead to proliferative, quiescent and necrotic regions, and similar gene expressions [1–3], which are unattainable and therefore not observed in 2D cell models. MDA-MB-231, a human epithelial triple-negative metastatic breast cancer cell line, is notorious for being difficult to grow in 3D [4]. Efforts to form 3D cellular structures with MDA-MB-231 often incorporate biologically based extracellular matrix (ECM) constituents, such as recombinant basement membrane (rBM) [1] or Matrigel [5, 6].

MDA-MB-231 lacks adequate capacity to form a stable structure. These cells display stellate morphologies when grown in 3D in the presence of an extracellular matrix, indicating a malignant phenotype [7, 8]. Matrigel, which promotes intercellular interactions for cell agglomeration [6], is derived from mouse Englebreth-Holm-Swarm tumor [9] and contains a mixture of ECM proteins and growth factors [6, 9]. It is used in numerous scaffold-based 3D culture models [10–14]. However, the batch-to-batch variability of endogenous components and uncontrolled matrix constituents [2, 15–17] in Matrigel limits the reproducibility of the structures that are formed. In addition, it does not appropriately represent a human microenvironment due to its murine-derived origin [18]. Regulating the formation of these 3D cellular structures is critical for a drug discovery process [6, 16, 19, 20].

The use of scaffold-free aggregations of tumor cells is an appropriate model for cancer research [2]. Adherent or anchorage-dependent cells cultured on an ultralow attachment (ULA) surface undergo spontaneous agglomeration, referred to as the forced floating [21] or liquid overlay technique [1, 6]. On a flat ULA surface, this forced floating results in the formation of numerous 3D cellular structures of variable dimensions [2], limiting the reproducibility of the desired 3D model. In a geometric-bottom well plate, cells are able to aggregate in numerous cavities and form 3D cell spheres (spheroids) with greater uniformity [3]. Although a high throughput is achieved in both cases, the isolation of a single 3D cellular structure poses difficulty. Alternatively, the use of a round-bottom ULA surface facilitates the formation of a single 3D cell structure in each well [22]. Here, only gravity is active in the formation of these structures. It follows that an externally applied force could concentrate the cells into a denser area and form a single 3D cellular structure on a flat ULA surface.

Magnetic printing is an engineering solution to create reproducible 3D cellular structures that can be used for *in vitro* cellular studies [23–28]. Here, using a unique bottom-up approach, 3D cellular assemblies can be formed by exploiting the magnetic properties of cells. Most mammalian cells are diamagnetic [29], i.e., they exhibit a repulsive magnetic force when in the presence of a magnetic field. This is also true of their culture medium, which is an aqueous solution of proteins, sugars, and nutrients to maintain their growth.

**Figure 1:**
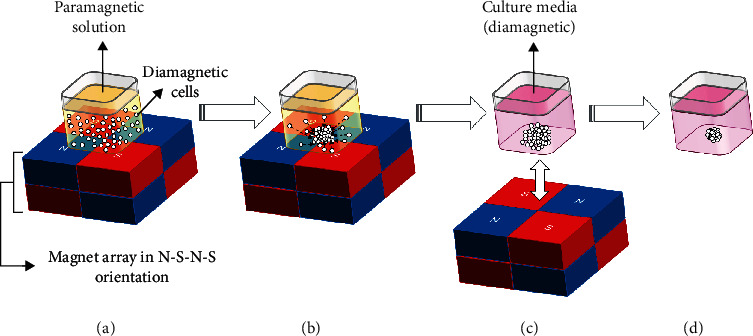
Schematic illustration of the magnetically assisted printing of 3D cellular structures. (a) Printing 3D cellular structures with magnetic assistance requires (1) a homogeneous suspension of cells in a liquid medium, (2) a magnetic susceptibility difference between the cells (diamagnetic) and the medium (paramagnetic), and (3) an applied inhomogeneous magnetic field. This is produced by a quartet of magnets in North-South-North-South (N-S-N-S) orientation. At the intersection of this quartet, there is a region of low magnetic field gradient. A physical well that is part of a standard 384- or 96-well plate is placed directly above this region. (b) The diamagnetic cells are displaced towards the center of the well. (c) After all cells have assembled in the center of the well and have had sufficient intercellular interactions to stabilize the cell agglomerate, the paramagnetic solution is replaced with regular culture media, and the magnetic field is removed. (d) The 3D cellular structure contracts as a result of continued intercellular interactions.

With the addition of a paramagnetic salt, such as gadopentatic acid (Gd-DTPA), the culture medium becomes paramagnetic. Within the appropriate exposure limits, Gd-DTPA (a magnetic resonance imaging (MRI) contrast agent (CA)) is potentially nontoxic. The addition of Gd-DTPA establishes a magnetic susceptibility difference between the diamagnetic cells and their surrounding paramagnetic liquid media and has been shown to effectively facilitate the displacement of cells in the presence of a magnetic field [23, 24, 26, 27]. In an inhomogeneous magnetic field, the suspended cells are displaced towards regions of lowest magnetic field strength to form a single 3D cellular cluster in a contactless, label-free manner within hours (Figure 1 and Video S1). We have previously demonstrated the rapid and highly reproducible formation of 3D MCF-7 and layer-on-layer cellular structures using this technique [23, 27].

*In vivo*, tumors may be composed of up to 80% stromal cells which include fibroblasts, adipose, endothelial and inflammatory cells, and a cocktail of different growth factors and enzymes [18]. Since the tumor microenvironment (TME) *in vivo* is highly regulated by the presence of stromal cells [30–32], soluble factors [33], and ECM [2, 34], an alternative to the addition of Matrigel or collagen is the coculture with fibroblasts [4, 18].

Fibroblasts are the most predominant cell type of connective tissue found in animals and actively produce and remodel the ECM [18, 35]. In addition, it has been shown that the activity of cancer-associated fibroblasts (CAFs) [36] or resident fibroblasts present in a TME [37] stiffen the TME containing tumor cells through the crosslinking of collagen; one of the most predominant proteins in ECM [38]. This results in the increase in fibroblast contraction and the number of focal adhesions [36], which are the interactions that anchor cells to ECM. Formation of a scaffold-free coculture tumor model also eliminates the need to employ laborious extraction techniques from a matrix, which are necessary for further downstream analyses [2, 39]. Fibroblasts have been used in previous studies to model the influence of stromal cells in a malignant tumor model [18, 30, 40, 41] and for conditioning culture medium to observe an increase of invasiveness [33] or motility [42] of breast cancer cell lines *in vitro*.

Since fibroblasts in a coculture have been shown to restore the formation of 3D cellular structures in comparison to 3D cellular structures formed from monotypic cell populations [30], the introduction of fibroblasts is expected to also accelerate the formation of a 3D cellular structure. We hypothesize that through secretion of their ECM components and contraction of the 3D cellular structure (1) fibroblasts act as a “glue” that supports the formation of 3D cellular structures, and (2) these structures are produced more rapidly and with higher reproducibility with magnetically assisted printing than through gravitational settling alone.

The effect of fibroblasts on the formation of 3D cellular structures of MDA-MB-231 cells is investigated for structures printed with magnetic assistance and through gravitational settling alone. An embryonic mouse fibroblast cell line, NIH/3T3, is mixed in various proportions into a population of MDA-MB-231 cells. The 3D cellular structures are printed with magnetic assistance on flat-bottom and those through gravitational settling on round-bottom ULA surfaces.

Initial experiments are performed to establish the exposure limits of Gd-DTPA to monotypic and coculture populations of MDA-MB-231 and fibroblast cells. Once the formation time is established, the maximum projected area and circularity, and cellular ATP of the 3D cellular structures are measured for 336 hours. These measurements provide insight into the use of Gd-DTPA as a reliable paramagnetic agent, independent of its effect on the formation of 3D cellular structures via magnetic assistance. Confocal imaging is used to visualize the self-assembling distribution of the individual cell lines at 3, 7, and 14 days postformation.

## 2. Results

### 2.1. Presence of Gd^3+^ in Monotypic 3D Cellular Structures to Assess the Susceptibility of 3D Cellular Structures to Gd-DTPA

As previously demonstrated, the printing of 3D cellular structures with magnetic assistance is facilitated by adding 25 mM Gd-DTPA to the cell culture medium [23–25]. During the formation of a 3D MCF-7 cellular structure, the limiting exposure time to Gd-DTPA was established by evaluating the relative changes in cell viability. Subsequently, the minimum time required to form a 3D cellular structure was determined. Cell viability was not significantly affected by exposure to 25 mM Gd-DTPA for 24 hours. The 3D structure was printed within 6 hours for specific conditions that include seeding cell density, magnet dimension, and well size [23].

Different cell lines, however, may have varying susceptibilities to different concentrations and chelates of Gd^3+^ ions that can have a toxic effect [43], which may also interfere with native intercellular interactions. Gd-DTPA has a short biological half-life of approximately 30 minutes [44]. When Gd^3+^ ions separate from its chelate, they pose a toxic threat through accumulation, e.g., in organ tissues [44].

To understand the influence of Gd-DTPA during the magnetically assisted printing of 3D cellular structures, the concentrations of Gd^3+^ ions within the 3D cellular assemblies during short-term incubation periods must first be measured. Retention of Gd-based MRI CAs are of particular interest to the radiology community since the realization of physiological complications following intravenous administration, such as nephrogenic systemic fibrosis (NSF) [45, 46].

**Figure 2:**
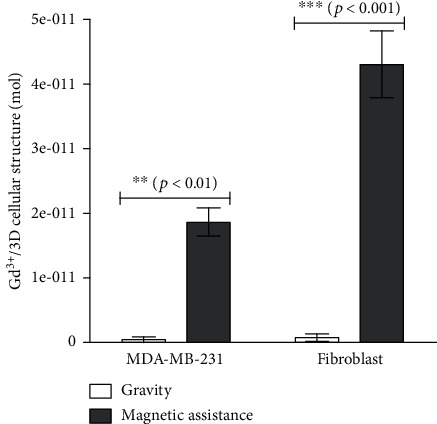
Presence of Gd^3+^ during formation of 3D cellular structures via magnetic assistance. The presence of Gd^3+^ ions within 3D cellular structures printed with magnetic assistance is observed in the two cell lines. A higher Gd^3+^ concentration is observed in 3D fibroblast structures (^∗∗∗^) than in the MDA-MB-231 structures (^∗∗^), as compared to their control samples formed through gravitational settling. Trace amounts of Gd^3+^ present in the control samples are attributed to instrument measurement sensitivity.

ICP-MS, a mass spectroscopy technique, is used to quantify the Gd^3+^ ions present in monotypic 3D cellular structures (MDA-MB-231 and fibroblast) printed with magnetic assistance (Figure 2). The 3D cellular structures printed with magnetic assistance are exposed to 25 mM Gd-DTPA in the presence of a magnetic field for 24 hours. Other 3D structures formed through gravitational settling, which do not require a paramagnetic medium or a magnetic field, are used as a control. These latter structures are therefore expected to have no Gd^3+^ ions present.

As shown in Figure 2, Gd^3+^ ions are present in 3D MDA-MB-231 or fibroblast cellular structures printed with magnetic assistance. Higher amounts of Gd^3+^ ions are present in fibroblast 3D structures (4.3 ± 0.9 × 10^−11^ mol/3D structure) than those composed of MDA-MB-231 cells (1.9 ± 0.4 × 10^−11^ mol/3D structure). Trace amounts of Gd^3+^ ions are also found in the 3D structures formed through gravitational settling for both MDA-MB-231 and fibroblast cells (4.4 ± 7.7 and 7.8 ± 9.8 × 10^−13^ mol/3D structure, respectively), but these amounts are close to the measurement limit of the instrument and thus considered insignificant.

Possible sources of Gd^3+^ retention in the 3D structures are entrapment within the ECM or cellular uptake. The internalization of Gd^3+^ ions in *in vivo* tissue and *in vitro* cellular structures can occur through passive diffusion [46] or through the displacement of ions due to similarities of the atomic radius size and competitive binding, such as in bone by replacing Ca^2+^ in hydroxyapatite [45].

Retention of Gd^3+^ has not been reported for breast cancer tissue composed of MDA-MB-231 cells, but its retention in kidney tissue has been measured to be 2.05 ± 0.17 ppm (~1.3 × 10^−9^ mol) two weeks following administration of clinically relevant dosages of Gd-DTPA [44]. The organ- or cell-specific toxicity of these levels of Gd^3+^ is unknown. In the future, to determine the subcellular presence of Gd^3+^ within the structures, scanning electron microscopy (SEM) energy-dispersive X-ray spectroscopy (EDS) [47], synchrotron radiation scanning transmission X-ray microscopy (SR-STXM) [48], or administration of a specifically membrane-permeable Gd-based CA detected by ICP-MS [49] may be used.

### 2.2. Effect of Gd-DTPA on Cell Viability in 2D Monotypic and Coculture Populations

The effect of Gd-DTPA on cell viability is evaluated on 2D monotypic and coculture populations using an MTT assay (Figure 2), which measures metabolic activity, an indication of cell viability. The coculture populations consist of 12.5, 25, and 50% fibroblasts. Exposure to higher Gd-DTPA concentrations over longer durations should be more toxic to cells than to lower concentrations, shorter exposure times, or a combination of the two.

**Figure 3:**
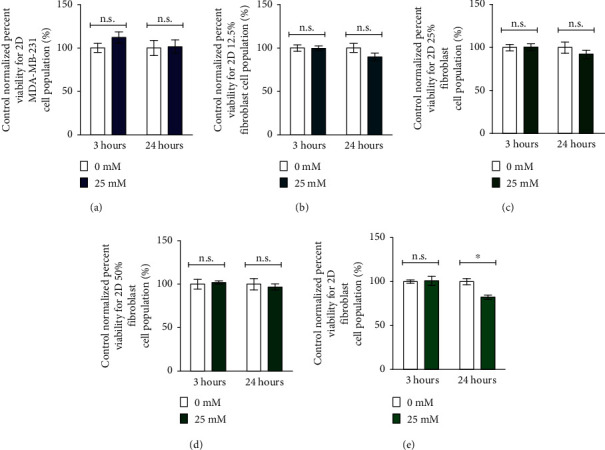
Control normalized percent viability. 2D cell populations of monotypic MDA-MB-231, fibroblast, and coculture populations are exposed to 25 mM Gd-DTPA for 3 and 24 hours. A 25 mM Gd-DTPA concentration does not influence the cell viability of (a) monotypic MDA-MB-231 and coculture cell populations composed of (b) 12.5%, (c) 25%, and (d) 50% fibroblast cells at 3 and 24 hours and (e) monotypic fibroblast cell populations at 3 hours. However, at 24 hours of exposure to 25 mM Gd-DTPA, the control percent viability for monotypic fibroblast cell populations decreases (^∗^).

Our previous investigations found exposure to 25 mM for 24 hours to be nontoxic to MCF-7 cells [23, 24]. Therefore, we continue to investigate the short-term cell viability of MDA-MB-231, fibroblast, and coculture populations at 25 mM Gd-DTPA for two incubation times. Each population is exposed to that concentration for 3 and 24 hours, and the cell viability is normalized to the corresponding Gd-DTPA-free (0 mM) control population. At 3 hours, none of the populations exhibit significant changes in cell viability. At 24 hours, while the monotypic MDA-MB-231 (Figure 3(a)) and coculture populations (Figures 3(b)– 3(d)) exhibit nonsignificant changes in cell viability, the monotypic fibroblast populations (Figure 3(e)) display a significant decrease.

This reduced viability of fibroblast cells agrees with the ICP-MS measurements that show increased susceptibility of fibroblast cells to Gd^3+^ ions; the presence of which is likely related to toxicity and thus affects cell viability. The reduced susceptibility of the monotypic MDA-MB-231 cell populations may be due to their inherent resistance to the salt. This may also be the case for cocultures where the resistance of the MDA-MB-231 cell population or its proportion overcomes the susceptibility of fibroblasts or their reduced metabolic inactivity in the presence of Gd-DTPA. The interaction between the two cell lines may also increase their overall resistance [30] towards Gd-DTPA.

Based on these results, to limit potential toxic effects of Gd-DTPA, the monotypic MDA-MB-231 and coculture cell populations are exposed to 25 mM Gd-DTPA for a maximum of 24 hours. However, fibroblasts are only exposed to 25 mM Gd-DTPA for a maximum of 3 hours.

### 2.3. Formation of Monotypic and Coculture 3D Cellular Structures to Assess the Influence of Cellular Composition and Method of Formation

Printing 3D cellular structures with magnetic assistance requires (1) a suspension of cells in a liquid medium, (2) a magnetic susceptibility difference between the cells (diamagnetic) and the medium (paramagnetic), and (3) an applied inhomogeneous magnetic field. After the 3D structures are printed, the magnetic field can be removed and the paramagnetic medium replaced with a usual cell medium [23]. However, if the paramagnetic cell medium is replaced with a Gd-free cell medium prior to sufficient intercellular interactions occurring, the cell agglomerate is readily disturbed and the morphology of the resulting 3D structure distorted. This can lead to the formation of numerous 3D daughter structures.

A 3D cellular structure is considered established when it contains cellular interactions [6] or retains its morphology following transfer into another well [50]. Hence, we compare the morphologies of the 3D structures before and after physical disturbances to the culture well in the form of cell medium washes (Figure S1), which are performed to remove Gd-DTPA. Here, formation is considered successful when a single 3D cellular structure is present following these necessary washes.

Due to the increased secretion of ECM factors *in vivo*, increasing the fibroblast proportion in the cell population *in vitro* is expected to decrease the time required to form a 3D structure. A summary of successful productions (%) of 3D cellular structures printed with magnetic assistance and formed through gravitational settling alone is provided in Table S1 and Table S2, respectively.

**Figure 4:**
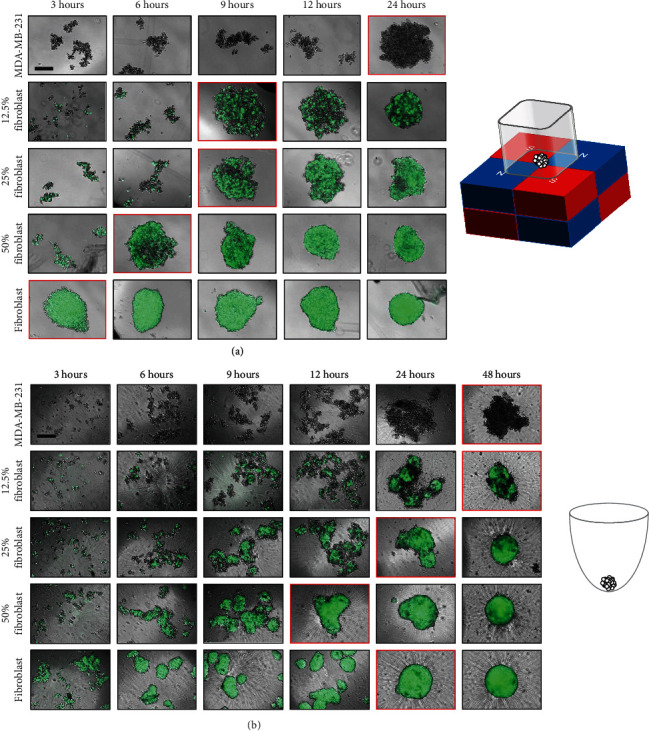
Formation of 3D cellular structures. 3D cellular structures composed of MDA-MB-231 (monotypic), 12.5, 25, and 50% fibroblast (cocultures) and fibroblast (monotypic) cells (a) printed with magnetic assistance and (b) formed through gravitational settling are imaged at 3, 6, 9, 12, 24, and 48 hours. A monotypic 3D structure containing MDA-MB-231 is formed at 24 hours with magnetic assistance; in contrast, a similar formation at 48 hours when gravity is used alone. As the proportion of fibroblast cells increases, the structure formation time decreases. Again, magnetically assisted printing reduces structure formation time as compared to formation under the influence of gravity alone for all cell populations. The panels with red borders indicate the time it takes to form a 3D cellular structure. The images are taken after the cell medium is replaced with fresh medium for both methods. The scale bar is equal to 100 *μ*m.

Each cell population requires a different incubation time to form a 3D cellular structure (Figure 4(a)). A single monotypic MDA-MB-231 3D cellular structure is confirmed at 24 hours when printed with magnetic assistance. Similar formation times have been reported for nongrowing aggregates [6] or loose aggregates created with rBM in a scaffold-free environment [1]. Otherwise, longer formation times are required to form robust 3D MDA-MB-231 structures [4, 51].

To further reduce the time for printing a 3D MDA-MB-231 structure, we introduce fibroblasts in various proportions. *In vivo*, fibroblasts are found throughout the body and act as scaffolds for other cells [35]. Therefore, fibroblasts can be used in an *in vitro* setting where they act as an adhesive that promotes intercellular interactions. As the proportion of fibroblasts increases in these binary cell mixtures, the time required to print a 3D cellular structure decreases.

The 12.5 and 25% fibroblast cell populations both produce 3D structures at 9 hours, while with 50% fibroblasts, a 3D structure is formed at 6 hours with magnetic assistance (Figure 4(a)). A monotypic fibroblast cell population forms a 3D cellular structure within 3 hours with magnetic assistance. These rapid formation times are within the exposure limits of Gd-DTPA determined from the MTT results. The results support our hypothesis that fibroblasts act as a “glue” that supports the formation of 3D cellular structures.

A round-bottom ULA plate that employs gravitational setting to form 3D structures is used as a control. The cell media is replaced at identical incubation periods used for magnetically assisted printing and images taken of their morphologies (Figure 4(b)). As expected, it takes longer to form a structure with gravity alone.

Monotypic MDA-MB-231 and fibroblast cell populations form 3D cellular structures at 48 and 24 hours, respectively. Similar to structures printed with magnetic assistance, introducing fibroblasts accelerates the formation of a 3D structure compared to one produced with a monotypic MDA-MB-231 cell population. When the fibroblast proportion introduced into an MDA-MB-231 cell population is doubled, the structure formation time decreases by half. 3D structures with 12.5% fibroblast are formed at 48 hours, those with 25% fibroblast at 24 hours, and with 50% fibroblast at 12 hours.

Observations made prior to 3D structure formation indicate that numerous 3D structures are present instead of a single principal cellular structure. Therefore, the delay in structure formation does not suggest an inability of the cells to establish intercellular interactions but is instead a consequence of their distances from each other as these interactions occur.

### 2.4. Growth of Monotypic and Coculture 3D Cellular Structures to Assess the Influence of Cellular Composition and Method of Formation

The long-term growth characteristics of 3D cellular structures, maximum projected areas, and circularities [6, 21, 23, 52, 53] are measured. Since the maximum projected structures are not perfect circles, it is not appropriate to use diameter as a metric [4]. Instead, the maximum projected area is used.

The maximum projected area of the 3D cellular structures is expected to decrease as a result of increasing cell agglomeration density before the area increases due to cell growth. This pattern of contraction followed by an increase in maximum projected size is typical for multicellular tumor spheroids (MCTS) [19], which are agglomerations of cancer cells in a scaffold-free environment. A size increase indicates that the 3D cellular structure is growing.

Circularity is defined as

(1) $$\begin{array}{c} \text{Circularity}=4\pi \left(\text{area}/\text{perimete}{\text{r}}^{2}\right), \end{array}$$

where a value of 1 indicates a perfect circle. Values smaller than 1 indicate a deviation from a perfect circle but do not provide information that describes the morphology of the structure.

**Figure 5:**
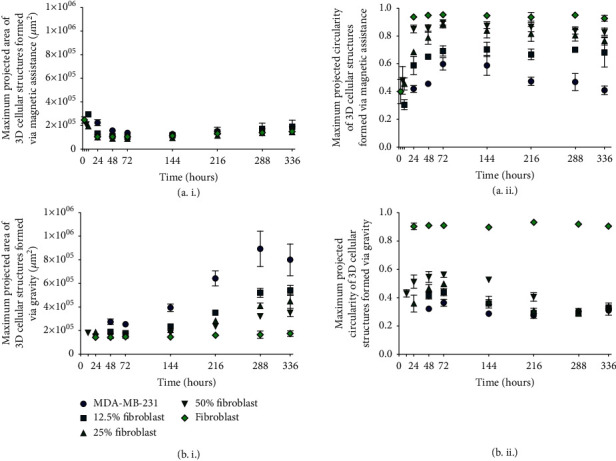
Growth of 3D cellular structures printed with magnetic assistance and formed under the influence of gravity alone. (i) Maximum projected area and (ii) circularity measurements of 3D cellular structures (a) printed with magnetic assistance and (b) formed through gravitational settling. The maximum projected areas of the monotypic MDA-MB-231 and 12.5 and 25% fibroblast coculture 3D structures printed with magnetic assistance decrease by 39, 64, and 55%, respectively, after their initial formation. The maximum projected areas of 3D structures containing a 50% fibroblast coculture and a monotypic fibroblast decrease by 51 and 57%, respectively, until 24 hours. These maximum projected areas are maintained until 72 hours. From 144 hours until 336 hours, the maximum projected areas increase slightly within experimental error. In contrast, the maximum projected areas of 3D cellular structures formed under the influence of gravity alone ((b), i) increase by 103, 130, 117, and 94% from 144 to 336 hours for monotypic MDA-MB-231 and 12.5, 25, and 50% fibroblast 3D structures, respectively. As the proportion of fibroblasts increases, structure circularity ((a), ii, and (b), ii) also improves. The legend in ((b), i), applies to graphs in panels ((a), i; (a), ii; and (b), ii).

Over a duration of 336 hours, 3D cellular structures printed with magnetic assistance (Figure 5(a)) and formed under the influence of gravity alone (Figure 5(b)) exhibit different growth behaviors. Maximum projected area measurements (Figure 5(a), i) for magnetically printed monotypic MDA-MB-231 3D structures and those containing 12.5 and 25% fibroblast cocultures decrease by 39, 64, and 55%, respectively, from their times of formation to 72 hours. Meanwhile, the maximum projected areas of 50% fibroblast-containing and monotypic fibroblast 3D structures decrease by 51 and 57%, respectively, from their times of formation until 24 hours and then maintained until 72 hours. An increase of 44, 61, 50, 50, and 30% in maximum projected areas is observed from 144 to 336 hours for monotypic MDA-MB-231, 12.5, 25, and 50% fibroblast-containing and monotypic fibroblast 3D structures, respectively. This suggests that there is cell growth in the 3D structures.

The maximum projected circularity (Figure 5(a), ii) of monotypic fibroblast 3D cellular structures is maintained between 3 and 336 hours. However, between their times of formation and 72 hours, the cocultured 3D structures become more circular, with maximum projected circularity increasing by 127, 94, and 86% for 12.5, 25, and 50% fibroblast-containing binary cell mixtures, respectively. From 144 to 336 hours, their maximum projected circularity is essentially maintained. As the proportion of fibroblasts in the binary mixture increases, the circularity also increases. Monotypic MDA-MB-231 3D structures become more circular between their time of formation (24 hours) and 72 hours with an increase of 43%. At 144 hours, their maximum projected circularity decreases by 30% at 336 hours.

Maximum projected areas for monotypic MDA-MB-231 and cocultured 3D cellular structures formed due to gravitational settling (Figure 5(b), i) remain unchanged from their times of formation (48, 48, 24, and 12 hours, for monotypic MDA-MB-231, and 12.5, 25, and 50% fibroblast cocultured 3D structures, respectively) until 72 hours. Afterward, these areas increase but do so more rapidly from 144 to 336 hours (where they exhibit 103, 130, 117, and 94% increases, respectively). As the fibroblast concentration increases in the cocultured structures, the increase in the maximum projected area becomes less rapid. Maximum projected area measurements for monotypic fibroblast 3D cellular structures do not change significantly from their times of formation (24 hours) until 336 hours.

The maximum projected circularity (Figure 5(b), ii) of a monotypic fibroblast 3D cellular structure formed through gravitational settling also does not change from its time of formation until 336 hours. The circularities of monotypic MDA-MB-231 and cocultured 3D cellular structures increase by 10, 6, 38, and 31% for monotypic MDA-MB-231, and 12.5, 25, and 50% fibroblast-containing 3D structures, respectively, from their times of formation until 72 hours. For monotypic MDA-MB-231, 12.5, and 25% fibroblast cocultured 3D structures, their maximum projected circularity decreases by 24, 34, and 38%, respectively, from 72 hours until 216 hours. For the 50% fibroblast coculture 3D structure, its maximum projected circularity decreases by 46% from 72 hours until 288 hours. As the proportion of fibroblasts increases, the circularity is higher and maintained until 336 hours, at which time the circularity between these various 3D structures is indistinguishable.

The growth characteristics observed from Figure 5 suggest that Gd-DTPA has a detrimental effect on 3D structures, preventing them from growing. Although fibroblast-containing aggregates appear to have higher amounts of Gd^3+^ per structure, the changes in their maximum projected areas are not significant. The MDA-MB-231 3D structures are affected by Gd-DTPA, but this is not suggested by the ICP-MS or MTT results. In contrast to the maximum projected area results, magnetically assisted printing improves the longer-term circularity of the structures as compared with those formed through gravitational settling. For all cases, the 3D structures are more circular when printed with magnetic assistance as compared to those with the same initial composition formed through gravitational settling.

A lytic process at the time of measurement is employed to quantify cellular ATP, which accesses all available ATP within the 3D cellular structure that may have otherwise been unaccounted for if an MTT assay were used. The cellular organization of metabolically active cells in the 3D structures can contribute to different ATP levels. Since the sizes of the 3D structures cannot be controlled, the measurements refer to total cellular ATP that is compared to a 40 pmol ATP reference and quantified with a standard curve calibration. As described in the manufacturer specifications (Promega), 40 pmol is the expected ATP recovery from a spheroid that has a diameter of 250 *μ*m, consistent with the sizes of the 3D structures in this investigation.

**Figure 6:**
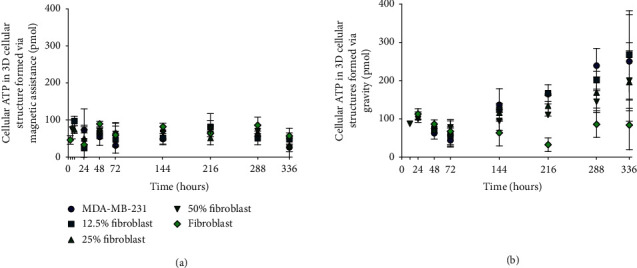
Cellular ATP of monotypic and coculture 3D cellular structures. Measurements are made from the time of formation until 336 hours for monotypic and cocultured 3D cellular structures (a) printed with magnetic assistance and (b) formed through gravitational settling. For monotypic and cocultured 3D structures (a) printed with magnetic assistance, cellular ATP does not change significantly within experimental error. Monotypic MDA-MB-231 and cocultures for structures (b) formed under the influence of gravity alone have a minimum cellular ATP level at 72 hours and monotypic fibroblast cellular structures at 216 hours. The legend in (a) applies to panel (b).

Cellular ATP measurements for 3D cellular structures are taken from the time of formation until 336 hours to determine the cell viability (Figure 6). Cellular ATP measurements for monotypic and coculture 3D structures printed with magnetic assistance (Figure 6(a)) are indistinguishable from one another for all measurements. This minimal change in cellular ATP is similar to the maximum projected area measurements (Figure 5(a), i).

Similar scaling of cellular ATP to the maximum projected area is observed for 3D structures formed with gravity alone (Figure 6(b)). The cellular ATP of monotypic MDA-MB-231 and cocultured 3D structures decreases from their times of formation by 30, 23, 20, and 10% until 72 hours, followed by an increase of 461, 375, 212, and 154% until 336 hours for monotypic MDA-MB-231, 12, 25, and 50% fibroblast cocultured structures, respectively. As the proportion of fibroblasts increases, the increase in cellular ATP for each measurement is less rapid. However, monotypic fibroblast 3D cellular structures exhibit very different behavior since the minimum cellular ATP occurs at 216 hours.

This also suggests that Gd-DTPA has a countering effect on the growth of the 3D structures. We previously observed a different phenomenon when a 2D monolayer of MDA-MB-231 cells was exposed to 0.1, 1, and 10 mM Gd-DTPA [25]. There, the cells did not appear to respond to Gd-DTPA, displaying similar characteristics to their control (0 mM) at 3 days (percent cell viable cell count) and at 6 hours (cell migration). This was conjectured to occur due to the absence of an estrogen receptor on these cells, which responds to Gd-DTPA as a xenoestrogen [25]. The complexity of a 3D structure, however, may introduce interactions with Gd-DTPA that interfere with regular cellular behaviour.

Although printing with magnetic assistance improves the reproducibility of 3D cellular structures, which is demonstrated by the better maximum projected area and circularity, this method could be improved further by replacing Gd-DTPA with a less cytotoxic paramagnetic agent. Alternatively, the present system can be optimized to limit cell exposure to Gd-DTPA by reducing the exposure time or salt concentration. To obtain a better understanding of the specific effect that Gd-DTPA has on the 3D structures, additional assays that target different metabolic processes should be performed, such as the resazuran reduction assay [54], response to drug toxicity [19], immunostaining [55], and profiling gene expression [7].

### 2.5. Self-Distribution of Individual Cell Lines within Cocultured 3D Cellular Structures to Assess the Significance of Cell Populations and Method of Formation

**Figure 7:**
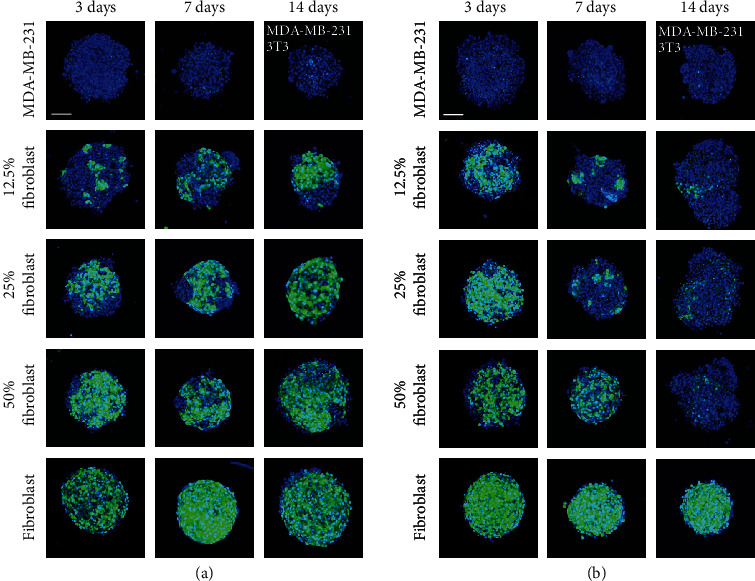
Visualization of self-distributing cell lines within coculture 3D cellular structures following formation. Confocal z-stack images showing regional distributions of MDA-MB-231 (blue) and fibroblast (green) cells in 3D cellular structures (a) printed with magnetic assistance and (b) formed through gravitational settling at 3, 7, and 14 days. The scale bar is equal to 100 *μ*m.

The segregation of cells within a 3D cellular structure is influenced by the cell lines used in the coculture [56]. We find that differences in the self-distributions of MDA-MB-231 and fibroblasts also depend on the method of formation (Figure 7). The long-term distributions are observed using confocal microscopy. For 3D structures printed with magnetic assistance (Figure 7(a)), numerous small regions containing fibroblasts (green) are observed at 3 days for an initial 12.5% fibroblast-containing mixture. This number of regions decreases at 7 days and continues to reduce when observed at 14 days. Similar behavior is observed for a 25% fibroblast-containing 3D structure, but there are smaller numbers of regions composed of MDA-MB-231 cells (blue) between 3 and 7 days. At 14 days, however, it becomes difficult to distinguish regions within the structures that contain primarily MDA-MB-231 cells or fibroblasts. The number of regions with primarily MDA-MB-231 cells appears to be unchanged in 50% fibroblast-containing 3D cellular structures. For all structures, single optical sections at *z* = 12 *μ*m and *z* = 24 *μ*m (Figure S2a) show that fibroblasts dominate within the mass, rather than form a capsule around the 3D cellular structure printed with magnetic assistance.

For 3D structures formed under the influence of gravity alone (Figure 7(b)), the proportion of fibroblasts appears to decrease over time and these cells concentrate toward the center of the structure, where they are surrounded by loosely aggregated MDA-MB-231 cells. Similar to 3D cellular structures printed with magnetic assistance, single optical sections at *z* = 12 *μ*m and *z* = 24 *μ*m (Figure S2b) indicate that fibroblasts concentrate more within of 3D cellular structures formed through gravitational settling.

The confocal microscopy analysis does not provide quantifiable data that can measure the physical space occupied by each cell line over time. The random orientations of cell constructs at the time of imaging can also affect the analysis. Although it may not be the case for the particular 3D cellular structures that we have produced, the difference in the doubling time between the two cell lines (36 hours for MDA-MB-231 and 20 hours for NIH/3T3, in 2D) should be considered when analyzing the relative proportions of each cell type over time.

In previous investigations, MDA-MB-231 have displayed endothelial-like morphologies when 3D cellular structures were cultured on Matrigel and injected into mice [5]. The ECM provided by fibroblasts may interact with tumor cells to prevent epithelium organization, instead of sustaining the formation of clusters [30]. The distribution of each cell line following formation might also be explained by the differences in their surface tensions [57], but this does not explain the observed differences between the two methods of forming 3D cellular structures with identical cell population identities.

Previous investigations of 3D *in vitro* cultures of human breast cancer and fibroblast cells report that the fibroblasts encapsulate breast tumor cells, maintaining their presence on the periphery of the spheroid, which is comparable to the organization of *in vivo* tumors [18]. We observe this only for the 3D cellular structures printed with magnetic assistance. For clinically relevant cells, the specific intercellular interactions may be observed using immunohistochemistry.

The differences between the two methods of formation, magnetically assisted printing and gravitational settling, suggest explanations for the corresponding maximum projected area and circularity, and cellular ATP measurements. Although their distribution changes over time when the 3D structures are printed with magnetic assistance, the constant presence of fibroblasts maintains a conserved maximum projected area and circularity. This correlates with the ATP measurements, which are also conserved and do not change significantly, and are independent of the composition of the monotypic or coculture 3D cellular structures. These observations suggest that printing through magnetic assistance is necessary to observe the long-term presence of both cell lines initially used. For 3D cellular structures formed through gravitational settling, the decrease in the presence of fibroblasts over time may explain the decrease in the maximum projected circularity of the structures.

Printing with magnetic assistance promotes intercellular interactions between monotypic cells that do not otherwise form 3D cellular structures readily. This is the case for the monotypic MDA-MB-231 cell population that is able to form reproducible 3D structures with magnetic assistance in 24 hours and through gravitational settling in 48 hours.However, the advantage of printing with magnetic assistance comes at the expense of limiting cell growth in the 3D structures that are produced.

## 3. Discussion

The unmet need for producing 3D cellular structures of MDA-MB-231 in a rapid high-throughput manner inspires unique approaches to overcome this challenge. This investigation explores how 3D cellular structures can be more rapidly printed with magnetic assistance than leveraging the influence of gravity alone. The results validate our hypothesis that (1) fibroblasts act as a “glue” that supports the formation of 3D cellular structures, and (2) the structures are produced more rapidly and with higher reproducibility with magnetically assisted printing than through gravitational settling alone.

We elucidate the differences between 3D MDA-MB-231 cellular structures printed with magnetic assistance and those formed under the influence of gravity alone. Fibroblasts are introduced to promote cell agglomeration. This is seen for both methods of forming 3D structures that contain human breast adenocarcinoma cells. We demonstrate that 3D structures composed of MDA-MB-231 cells can be printed with magnetic assistance within 24 hours without using a scaffold or matrix to promote cell agglomeration. This incubation time is not observed in literature without additional reagents.

To avoid affecting the magnetic susceptibility of the cells by labeling with [18] or internalization of [58] a magnetic particle, the magnetic susceptibility of the medium is changed by adding a magnetic salt, creating a paramagnetic solution [59]. A lower magnetic susceptibility causes the cells to be displaced towards regions of the lowest magnetic field strength [60].

Printing with magnetic assistance (also termed the magneto-Archimedes effect [61]) allows label-free manipulation of nonmagnetic cells. A magnetic buoyancy force is introduced by applying a magnetic field to a system where there is a difference in the magnetic susceptibility of a suspended analyte and its surrounding suspension medium [62]. The magnetic force acting on a cell,

(2) $$\begin{array}{c} F_{m}=\left[\frac{\left(\chi_{c}-\chi_{m}\right)}{\mu_{0}}\right]V_{c}\nabla {\left|B\right|}^{2}, \end{array}$$

where *χ*_*c*_ and *χ*_*m*_ denote the magnetic susceptibilities of the suspended cells and the suspension medium, respectively, *μ*_0_ the permeability of free space, *V*_*c*_ the volume of a cell, and ∇|**B**|^2^ the gradient of the square of the magnetic field. This is a high throughput method, e.g., realized by forming an array of magnets in an alternating North-South-North-South orientation and aligning a standard 96- or 384-well plate so that the intersection of the four magnets is centered to each well (Figure 1 and Video S1). Each well containing a suspension of diamagnetic cells in the paramagnetic medium will form a 3D cellular structure as described in Materials and Methods.

Although NIH/3T3 cells are of animal origin, their intended use in this study is to evaluate the influence of fibroblasts on the formation of 3D cellular structures for cell types which are difficult to cohere without using additional reagents. Such a use of cell lines from different species has been used in previous coculture models [33, 63]. Since human and mouse fibroblasts behave similarly in terms of their ability to produce ECM proteins (such as collagen) [64–66], the conclusions of our study demonstrate the capability of magnetically assisted printing in this coculture model.

For disease-specific modeling in a clinical setting, where it will be necessary to use human-derived cell lines or primary cells, this technique has the potential to form different shapes which may be more physiologically relevant than a spherical model. Improving the formation of 3D cellular structures by printing them with magnetic assistance has applications for tissue engineering, drug discovery, and lab-on-chip devices.

## 4. Materials and Methods

### 4.1. Cell Culture

Human MDA-MB-231 (American Type Culture Collection (ATCC), USA) and mouse green fluorescent protein- (GFP-) transfected (GFP^+^) NIH/3T3 (ATCC, USA, code CRL-1658) cells were both gifts obtained from colleagues. GFP^+^ NIH/3T3 cells were used for all investigations that mentioned fibroblast or NIH/3T3 cells. Both cell lines were maintained in Dulbecco Modified Eagle's medium (DMEM, Life Technologies, catalog number 12800-082) containing 10% fetal bovine serum (FBS, cat. no. 12484028). Phosphate buffered saline (PBS, cat. no. 10010023) and Trypsin-EDTA (0.25%), phenol red (cat. no. 25200056), used for cell culture maintenance were purchased from Life Technologies, Canada. The cells were maintained at standard culture conditions (37°C, 5% CO_2_ in a humidified environment).

### 4.2. Synthesis of Paramagnetic Gd-DTPA Medium

Gadopentetic acid (Gd-DTPA) salt hydrate purchased from Sigma-Aldrich, Canada (cat. no. 381667) was used to make a 200 mM Gd-DTPA solution in the culture medium. Immediately after dissolution, 1 M sodium hydroxide (NaOH, Alfa Aesar, cat. no. A16037) was then added dropwise to adjust the pH to physiologic levels, to approximately 7.4 ± 0.2. Contents were constantly mixed on a stir plate as Gd-DTPA and NaOH were added. Subsequent dilutions were prepared with the culture medium.

### 4.3. Preparation of 3D Cellular Structures

3D cellular structures printed with magnetic assistance were prepared by seeding a cell concentration of 5000 cells/80 *μ*L of 25 mM Gd-DTPA culture medium per well into a 384-well flat-bottom ULA plate (Corning, product no. 4588). A quartet of 4.5 × 4.5 × 4.5 mm N52 magnets (Zigmyster Magnets) was arranged into an N-S-N-S orientation and placed directly underneath each well. 3D cellular structures formed through gravitational settling were prepared by seeding a cell concentration of 5000 cells/80 *μ*L of regular, Gd-DTPA-free (0 mM Gd-DTPA) culture medium per well into a 384-well U-bottom ULA plate (SBio, cat. no. MS-9384UZ). At the end of the specified exposure time during magnetically assisted printing, the paramagnetic medium was removed through a series of washes with regular, Gd-DTPA-free culture medium (0 mM Gd-DTPA). This was also performed for 3D cellular structures formed through gravitational settling, to determine the appropriate formation time. Half of the culture medium present in the 3D cellular structure samples was removed and replaced with fresh culture medium every 3 days.

### 4.4. Inductively Coupled Plasma Mass Spectrometry (ICP-MS)

MDA-MB-231 and fibroblast 3D cellular structures printed with magnetic assistance and formed through gravitational settling were prepared and pooled together after 24 hours. The pooled samples were then centrifuged, and the supernatant was removed. Each sample was washed with PBS five times to remove residual cell culture medium, which also contained Gd-DTPA for the 3D cellular structures formed via magnetic assistance. A final wash with ultrapure water was performed to dilute the salts present in PBS, which could interfere with ICP-MS measurements. The samples were stored at -20°C until the ICP-MS apparatus (Agilent 7700 series) was ready. When ready, the samples were digested with concentrated nitric acid and measured to detect Gd^3+^ (157 atomic mass units) with helium for plasma generation.

### 4.5. MTT Assay Analysis for Viability of 2D Cell Monolayers

For each cell population, 1000 cells were plated into a 96-well tissue culture-treated plate at 0 hours. Duplicate samples were prepared and exposed to 100 *μ*L of either 0 or 25 mM Gd-DTPA in the culture medium for each time of measurement. MTT reagent (3-(4,5-dimethylthiazol-2-yl)-2,5-diphenyltetrazolium bromide, Invitrogen, Canada, cat. no. M6494) was made into a 5 mg/mL solution in PBS. At 3 and 24 hours, the culture medium was removed from the samples and replaced with fresh culture medium. 10 *μ*L of MTT solution was added to each sample and incubated for 3 hours at standard culture conditions. Following incubation, 85 *μ*l of the solution was removed and 50 *μ*L dimethyl sulfoxide (DMSO, Sigma-Aldrich, Canada, cat. no. D4540) was added. The samples were again incubated at standard condition, for 10 minutes. The plate was then shaken, and the absorbance was read at 570 nm using the Tecan Infinite M200 plate reader. For each incubation period, the absorbance was normalized to the 0 mM (Gd-DTPA-free) control sample to measure the relative percent viability of cells exposed to 25 mM Gd-DTPA.

### 4.6. Size Measurements

For each monotypic and coculture 3D cellular structure printed with magnetic assistance or formed through gravitational settling, images were taken using a Carl Zeiss Axio Observer Z1 microscope: at the time of formation, 24 hours (if applicable), 48 hours (if applicable), 72, 144, 216, 288, and 336 hours. Images were analyzed with Fiji (ImageJ) imaging software, and maximum projected area and circularity measurements were obtained.

### 4.7. Measurements of Cellular ATP

For each monotypic and cocultured 3D cellular structure printed with magnetic assistance or formed under the influence of gravity alone, samples in 25 *μ*L of their medium were transferred into a white U-bottom 384-well plate (SBio, cat. no. MS-9384WZ). 25 *μ*L of CellTiter®-Glo 3D Viability Assay (Promega, part no. G9681) was added to lyse the 3D cellular structures and access cellular adenosine triphosphate (ATP). The samples were then shaken at 3 mm amplitude for 5 minutes and left to incubate at room temperature for an additional 25 minutes. Finally, the samples were read by luminescence with 1-second attenuation time. For all readings, a 40 pmol sample was used as a reference and normalized to a standard curve to quantify the relative luminescence units (RLU).

### 4.8. Confocal Microscopy

For each monotypic and coculture 3D cellular structure printed with magnetic assistance or formed through gravitational settling, three replicate samples were prepared at time equal to 0. On days 2, 6, and 13, 4′,6-diamidino-2-phenylindole (DAPI) blue fluorescent nucleic acid stain was added to each sample, staining all nuclei, both MDA-MB-231 and fibroblast, blue. After 24 hours of incubation at standard conditions, the 3D cellular structures were imaged on days 3, 7, and 14, respectively, using a Nikon A1R confocal microscope. A z-stack with a step size of 2.4 *μ*m was acquired from the bottom-most focused plane to approximately half the thickness of the 3D cellular structures. 2D reconstructed images were formed by taking the maximum intensity pixels at each stack for blue and green fluorescent channels. MDA-MB-231 cells were identified by their nucleus (blue) while fibroblasts were identified by an overlay of their nucleus and inherent GFP^+^ fluorescence (blue and green, respectively). Excitation and emission wavelengths of 395/509 and 358/461 were used for GFP and DAPI, respectively.

### 4.9. Statistical Analysis

Three biological samples of 3D cellular structures printed with magnetic assistance and formed through gravitational settling were prepared for ICP-MS measurements, each with ≥64 technical replicates. Pooled biological samples that were below the method reporting limit (MLR) were below the sensitivity limit of the instrument and therefore assigned a value of 0. ICP-MS results were analyzed by the standard error of the mean (SEM) of the pooled biological samples. A two-way analysis of variance (ANOVA) with Bonferroni post test was performed. A *p* value of <0.01 had two-star significance (^∗∗^), while a *p* value of <0.001 had three-star significance (^∗∗∗^).

Three biological samples of 2D monolayers for each cell population were prepared for MTT analysis, with six technical replicates for control and 25 mM Gd-DTPA samples, at each time of measurement, i.e., 3 and 24 hours. MTT results for 25 mM Gd-DTPA were control-normalized to their respective 0 mM Gd-DTPA sample and analyzed by SEM. A two-way ANOVA with Bonferroni post test was performed. A *p* value of >0.05 had no statistical significance, while a *p* value of <0.05 had one-star significance (^∗^).

Two biological samples of 3D cellular structures printed with magnetic assistance and six biological samples of 3D cellular structures formed through gravitational settling were prepared for the determination of the formation of 3D cellular structures. For each sample, four technical replicates were used at each specified time.

Two biological samples of 3D cellular structures printed with magnetic assistance and three biological samples of 3D cellular structures formed through gravitational settling were measured for their maximum projected area and circularity. For each sample, four technical replicates were used and SEM was calculated.

Three biological samples of 3D cellular structures printed with magnetic assistance and three biological samples of 3D cellular structures formed through gravitational settling were prepared for the measurement of cellular ATP. For each sample, four technical replicates were used and SEM was calculated.

Three biological samples were prepared for 3D cellular structures printed with magnetic assistance and three biological samples of 3D cellular structures formed through gravitational settling were prepared and imaged for confocal analysis. Representative images were selected for qualitative analysis of the cell distribution within the 3D cellular structures following formation.

All statistical analysis was performed using GraphPad Prism software with a 95% confidence interval.

## Supplementary Materials

Supplementary 1 Video S1: formation of 3D cellular structures through magnetic assistance.

Supplementary 2 Figure S1: morphology of 3D cellular structures (a) printed with magnetic assistance and (b) formed through gravitational settling before and after washes with the medium to remove Gd-DTPA and prevent overexposure to its ions. Figure S2: single optical sections at *z* = 12 *μ*m and *z* = 24 *μ*m for 3D cellular structures (a) printed with magnetic assistance and (b) formed through gravitational settling. Table S1: fraction of successful formation of 3D cellular structures with different percent cell populations and exposure times (hours) for printing with magnetic assistance. Table S2: fraction of successful formation of 3D cellular structures with different percent cell populations and exposure times (hours) for structures formed through gravitational settling.
