# Supplementary figures and images for: Fibroblasts Accelerate Formation and Improve Reproducibility of 3D Cellular Structures Printed with Magnetic Assistance
Source: Research (Wash D C). 2020 Jul 23;2020:3970530. doi: 10.34133/2020/3970530 (PMC7395227; doi:10.34133/2020/3970530)

## Slide 1
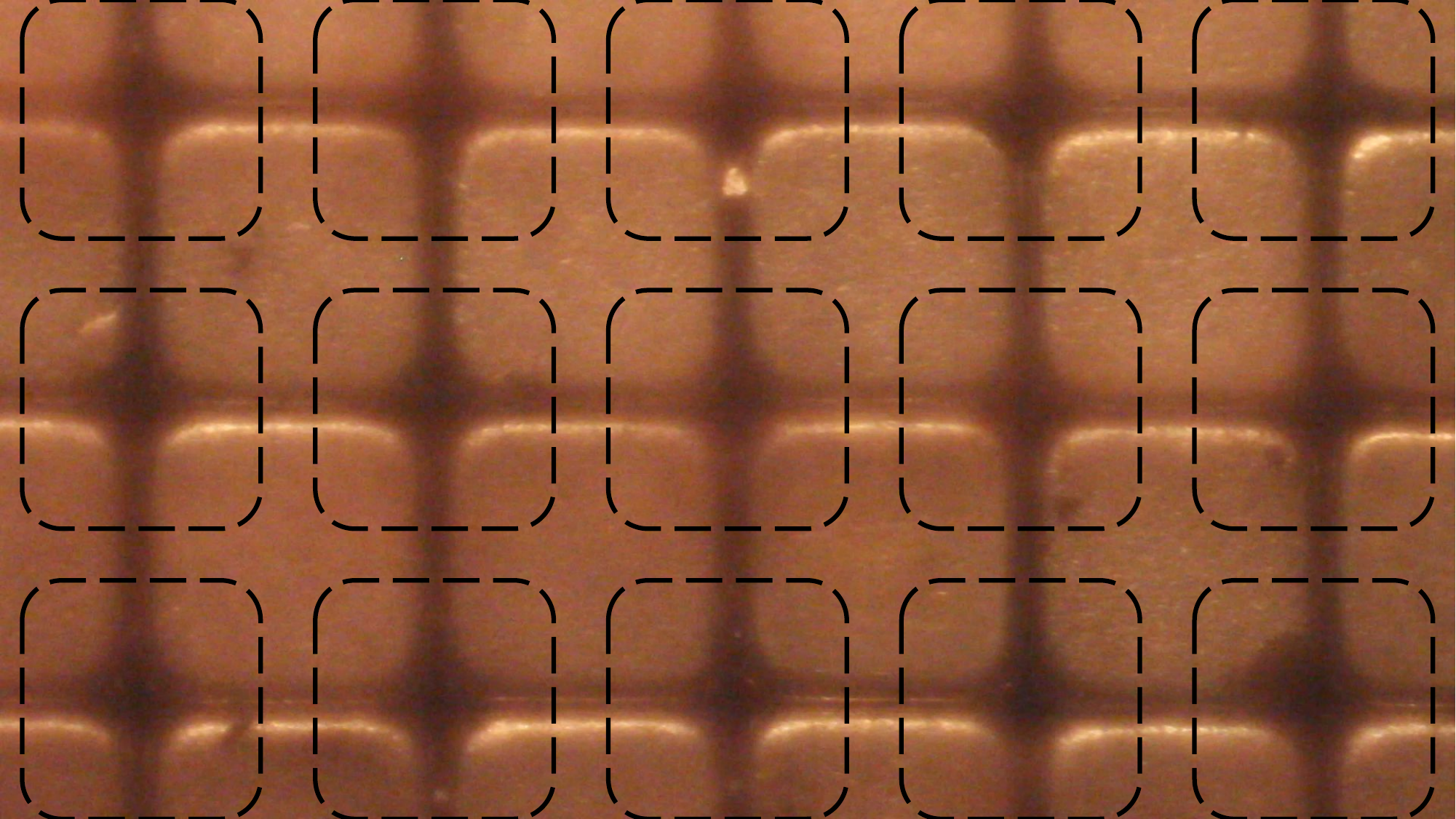

Supplement: Supplementary 1 — Video S1: formation of 3D cellular structures through magnetic assistance. [file 3970530.f1.pptx]
