# Supplementary material for: Fibroblasts Accelerate Formation and Improve Reproducibility of 3D Cellular Structures Printed with Magnetic Assistance
Source: Research (Wash D C). 2020 Jul 23;2020:3970530. doi: 10.34133/2020/3970530 (PMC7395227; doi:10.34133/2020/3970530)
Supplement: Supplementary 2 — Figure S1: morphology of 3D cellular structures (a) printed with magnetic assistance and (b) formed through gravitational settling before and after washes with the medium to remove Gd-DTPA and prevent overexposure to its ions. Figure S2: single optical sections at z = 12 μm and z = 24 μm for 3D cellular structures (a) printed with magnetic assistance and (b) formed through gravitational settling. Table S1: fraction of successful formation of 3D cellular structures with different percent cell populations and exposure times (hours) for printing with magnetic assistance. Table S2: fraction of successful formation of 3D cellular structures with different percent cell populations and exposure times (hours) for structures formed through gravitational settling. [file 3970530.f2.docx]

**Supplementary Materials**

**
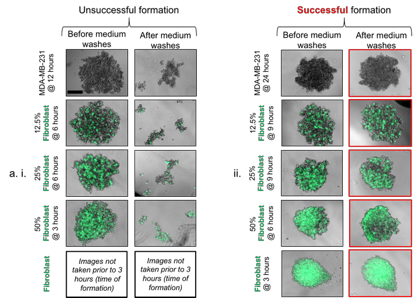
**

**
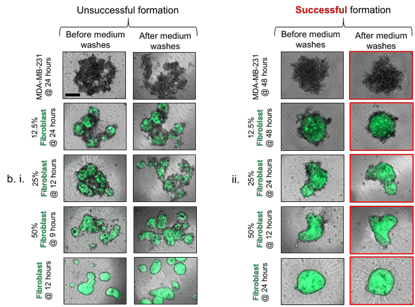
**

**Figure S1.** Morphology of 3D cellular structures (**a**) printed with magnetic assistance and (**b**) formed through gravitational settling before and after washes with the medium to remove Gd-DTPA and prevent overexposure to its ions. Cells require time to form a 3D cellular structure that will cohere. Prior to this coherence, (**i**) cells still form single 3D cellular structures but this disintegrates during washing, which disturbs their morphology. After the structure coheres, (**ii**) changes in the medium by washing are unable to overcome intercellular interactions and disturb the single 3D cellular structures. The scale bar is equal to 100 μm.


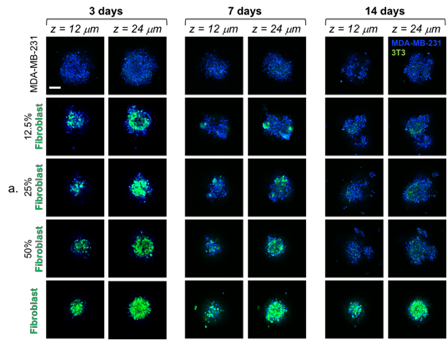


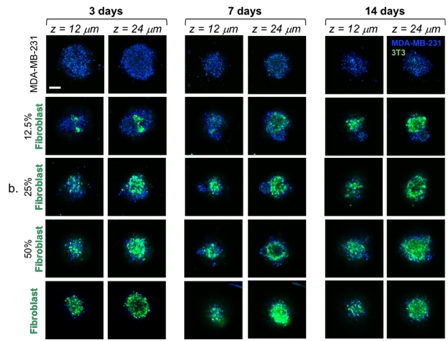


**Figure S2**. Single optical sections at z = 12 μm and z = 24 μm for 3D cellular structures (**a**) printed with magnetic assistance and (**b**) formed through gravitational settling. Although the presence of fibroblasts diminishes over time for 3D cellular structures formed through gravitational settling, both methods of formation indicate that fibroblasts concentrate within the structures, rather than form a capsule.

**Table S1**. Fraction of successful formation of 3D cellular structures with different percent cell populations and exposure times (hours) for printing with magnetic assistance. Interruptions were introduced to the culture well to remove the paramagnetic medium. A cut-off of 75% is used to determine the appropriate time required for successful formation.

| **Exposure**  **time**  **Cell**  **population** | **3** | **6** | **9** | **12** | **24** |
| --- | --- | --- | --- | --- | --- |
| **MDA-MB-231** | *0* | *0* | *29* | *38* | *100* |
| **12.50%** | *0* | *13* | *88* | *88* | *100* |
| **25%** | *0* | *63* | *100* | *100* | *100* |
| **50%** | *50* | *75* | *100* | *100* | *100* |
| **Fibroblast** | *100* | *100* | *100* | *100* | *100* |

**Table S2**. Fraction of successful formation of 3D cellular structures with different percent cell populations and exposure times (hours) for structures formed through gravitational settling. Interruptions were introduced to the culture well to replace the cell medium. A cut-off of 75% is used to determine the appropriate time required for successful formation.

| **Exposure**  **time**  **Cell**  **population** | **3** | **6** | **9** | **12** | **24** | **48** |
| --- | --- | --- | --- | --- | --- | --- |
| **MDA-MB-231** | *0* | *0* | *0* | *0* | *41* | *86* |
| **12.50%** | *0* | *0* | *0* | *0* | *61* | *92* |
| **25%** | *0* | *0* | *21* | *72* | *80* | *92* |
| **50%** | *42* | *56* | *68* | *78* | *92* | *100* |
| **Fibroblast** | *48* | *56* | *58* | *69* | *81* | *88* |
